# “A patient like me” – An algorithm-based program to inform patients on the likely conditions people with symptoms like theirs have

**DOI:** 10.1097/MD.0000000000017596

**Published:** 2019-10-18

**Authors:** Gideon Koren, Daniel Souroujon, Ran Shaul, Allon Bloch, Ariel Leventhal, Jason Lockett, Varda Shalev

**Affiliations:** a Maccabi-Kahn Institute of Research and Innovation; b Sackler School of Medicine Tel Aviv University; c Ariel University, Israel; d K Health, New York, NY; e Integrity Family Care, Huntsville Alabama.

**Keywords:** algorithm, diagnosis, symptoms

## Abstract

To date, consumer health tools available over the web suffer from serious limitations that lead to low quality health- related information. While health data in our world are abundant, access to it is limited because of liability and privacy constraints.

The objective of the present study was to develop and evaluate an algorithm-based tool which aims at providing the public with reliable, data-driven information based and personalized information regarding their symptoms, to help them and their physicians to make better informed decisions, based on statistics describing “people like you”, who have experienced similar symptoms.

We studied anonymized medical records of Maccabi Health Care. The data were analyzed by employing machine learning methodology and Natural Language Processing (NLP) tools. The NLP tools were developed to extract information from unstructured free-text written by Maccabi's physicians.

Using machine learning and NLP on over 670 million notes of patients’ visits with Maccabi physicians accrued since 1993, we developed predictors for medical conditions based on patterns of symptoms and personal characteristics.

The algorithm was launched for Maccabi insured members on January 7, 2018 and for members of Integrity Family Care program in Alabama on May 1, 2018.

The App. invites the user to describe her/ his main symptom or several symptoms, and this prompts a series of questions along the path developed by the algorithm, based on the analysis of 70 million patients’ visits to their physicians.

Users started dialogues with 225 different types of symptoms, answering on average 22 questions before seeing how people similar to them were diagnosed. Users usually described between 3 and 4 symptoms (mean 3.2) in the health dialogue.

In response to the question “conditions verified by your doctor”, 82.4% of responders (895/1085) in Maccabi reported that the diagnoses suggested by K's health dialogues were in agreement with their doctor's final diagnosis. In Integrity Health Services, 85.4% of responders (111/130) were in agreement with the physicians’ diagnosis.

While the program achieves very high approval rates by its users, its primary achievement is the 85% accuracy in identifying the most likely diagnosis, with the gold standard being the final diagnosis made by the personal physician in each individual case. Moreover, the machine learning algorithm continues to update itself with the feedback given by users.

## Abstract 2

Supplemental Digital Content is available in the text

## 1 Introduction

Every human encounters health problems, with consequences ranging from discomfort, to impairment of quality of life, to posing a serious threat. The internet is a natural channel to seek information complementary to established health care systems, whether as a preliminary information source, as a second opinion, or in cases where medical assistance is not available. However, to date consumer health tools available over the web suffer from serious limitations that lead to low quality health-related information. While health data are abundant, access to them is limited because of liability and privacy constraints.^[1]^ Medical content is often hard to understand, and its presentation is often misleading and may do more harm than good to the average person, leading to either unnecessary alarm or unjustified comfort.

In a recent publication on its official blog, Google stated that “health content on the web can be difficult to navigate, and tends to lead people from mild symptoms to scary and unlikely conditions, which can cause unnecessary anxiety and stress.”.^[2]^

To gain preliminary understanding of current patient behavior, we conducted a survey of the public about their search behaviors for on- online medical information, and their existing perceptions of this information's accuracy and reliability (Appendix). The survey, conducted in December 2017 based on a random sample of 500 adults, indicated that the public, searching for a great deal of medical information online, believes that it is unreliable, and nonetheless often relies on it.

The main disadvantages identified by respondents were that the data were not personally suited to their case, were insufficiently professional, the information caused stress and anxiety, and it generally presented severe or extreme options.

In numerous cases, such searches drove action: One in 10 survey responders took medications or began medical treatment on their own initiative following an online search.

These findings are concerning as they highlight the depth of the problem and the degree of helplessness that the public feels when coming across a medical issue.

The objective of the present study was to develop and evaluate an algorithm-based tool that provides the public with a reliable, data-driven information, based on personalized information regarding their symptoms. The tool is intended to help them and their physicians to make better informed decisions, based on how “people like you”, have experienced similar symptoms.

## 2 Subjects and methods

Maccabi Healthcare Service (MHS) is Israel's second largest health fund, serving over 2 million citizens. We maintain central computerized databases containing demographic and medical data, including physicians’ visits, hospitalizations, drug purchases (all prescriptions and part of OTC drugs), laboratory data and physician visits.^[3]^ It includes over 400 million notes from patients’ visits with Maccabi physicians accrued since 1993.

Our objective was to train the computer to understand this rich repository of health data and build a tool that consumers could use to easily learn about their health by referencing similar cases from people who share their demographic information, past medical information, and history of present illness. The study design complied with the Standards for Reporting Diagnostic accuracy studies (STARD), This study was approved by Assuta hospital Research Ethics Board in Tel Aviv.

### 2.1 Machine learning and natural language processing

The data were analyzed by employing machine learning methodology and Natural Language Processing (NLP) tools.^[4–8]^ Using machine learning and NLP on over 670 million notes of patients’ visits with Maccabi physicians accrued since 1993, we developed predictors for medical conditions based on patterns of symptoms and personal characteristics.

#### 2.1.1 Step 1

As a starting point, we studied anonymized medical records of MHS by running aggregated statistical tests and measurements on sections of the anonymized data, such as certain age groups or with certain medical symptoms. K Health has developed proprietary NLP tools to extract information from unstructured free-text written by Maccabi's physicians. Our NLP algorithms had to overcome the challenge of extracting symptoms and attributes of symptoms from doctor visit text notes, understand negative symptoms (for example: “the patient has a headache for 3 days, no fever”). In addition to proprietary tools, we also used standard tools such as Word2Vec to identify similar objects, and TD-IDF to find features which are highly linked to specific conditions.

#### 2.1.2 Step 2

Once we turned unstructured notes into structured data, the machine began to understand how symptoms present themselves, according to the patient's own language captured in their doctor's notes. The machine began to recognize patterns of how symptoms present differently in different adults 18 to 85 years of age, according to their age and gender. More than 10,000 medical notes were manually tagged and annotated, first to allow our Auto-tagging tools to learn and train on how a medical domain expert would tag these records, and second to test its accuracy after each and every run.

#### 2.1.3 Step 3

Subsequently, we developed classification (machine learning) algorithms to determine the likelihood of medical conditions based on patterns of symptoms and personal characteristics. At this point, the machine had learned how to recognize the correlation between groups of symptoms and a particular medical condition taking into account the user's demographics, past medical information and history of present illness. We employed multiple classification algorithms, ranging from a simple Bayesian network classifier, through logistic regression models, and random-forest based classifications (e.g., XG Boost) and Neural networks. These classifiers are binary and are trained on multi label records to be able to produce multi label results. They all work by receiving a vector of features comprised of the patient's past medical history, together with the history of the present illness to produce an output vector of labels which represents the distribution of conditions that the cluster of people like him would have received by Maccabi physicians.

#### 2.1.4 Step 4

From there, we used a machine learning process to deconstruct the doctor notes into a set of symptom attributes and values. Hence, not only does the machine learning recognizes, for example, headache, but it understands duration, severity, location, quality, and other factors that distinguish between different kinds of headache and symptoms that commonly concur with them. Thus, we built a medical ontology that represents tens of thousands of symptoms and attributes such as ”a headache, for 3 days, radiates to the arm and accompanied by dizziness." This step was performed manually in the beginning, and with time, our NLP tools learned how to recognize ∗new∗ connections between already defined symptoms with already defined attributes.

#### 2.1.5 Step 5

At this point, however, there was no way for a user to interact with the machine; hence, we designed a machine-based conversation method that mimics the conversation a doctor has with a patient. The machine determines in real time what is the best next question it should ask in order to understand the user's symptoms, rule out serious conditions, and obtain a complete understanding of the user's illness. With every question, the machine refined the most accurate cohort of similar people with a similar set of symptoms (i.e., People Like Me (PLM)).

When the machine has reached a level of confidence in understanding the user's symptoms, it ends the conversation and shows the user results based on the cohort of PLM cases with similar demographics, past medical information and history of present illness. Out of millions of medical cases, we present to them in aggregate the distribution of the most common diagnoses and treatments experienced by the PLM cohort. Visually we present to the user the PLM medical cohort's path to treatment, including the various conditions with which they were diagnosed, along with the full course of action of the cohort, which includes the types of medical professionals seen, tests ordered, medications prescribed, and expected recovery time.

#### 2.1.6 Step 6

Finally, we created a follow up conversation, which asks the user whether they saw a doctor and what was their eventual diagnosis and treatment. Thus we created a closed self-learning loop where the machine learning process automatically updates its model, conversation method and conditions presented based on physician-verified outcomes.

### 2.2 Statistical analysis:

Descriptive statistics were employed to describe the response to the use of the App. and different characteristics of the study group.

Diagnostic accuracy was defined as the percentage of diagnoses by the App that agreed with the preferred final diagnosis of the physician caring for the patient. Statistical methods involved in machine learning have been previously described by our group.^[7]^

## 3 Results

### 3.1 Case

A healthy 26 year old woman in the 13th week of her pregnancy, employed as a wedding master, finished the management of a wedding ceremony at 21:15, when she started experiencing abdominal pain. After 2 hours of increasing symptoms she turned to the K app on her smartphone. The app started by prompting questions about her symptoms and continued checking for further symptoms. By the end of a 3-minute process, after 22 questions, the woman received a result based on the learned outcome of 45,000 similar cases. The top choice was acute appendicitis. The woman was rushed by her husband to a nearby emergency room and 3 hours later she had surgery for acute appendicitis.

The K Health App was launched for MHS- insured members on January 7, 2018 and for members of Integrity Family Care in Alabama on May 1, 2018 and very recently became available in the US nationwide.

The App. invites the user to describe her/ his main symptom or several symptoms, and this prompts a series of questions along the path developed in the algorithm, based on the analysis of 70 million patients’ visits to their physicians.

By September 1, 2018 K Health surpassed 180,000 downloads, at a level of 1500 to 2000 a day. By September 1, 2018 K had more than 200,000 health dialogues that reached results. Over 80% of users who started a health dialogue continued and answered 20+ questions spending 3 minutes to receive results. In Integrity Family Care, Alabama, 30% of patients have been using the app by September 1, 2018.

Fifty one percent of users were men and 49% women. People were using the App throughout the day and night, with 12:00 to 22:00 being the busier hours.

Users started dialogue with 225 different types of symptoms, answering on average 22 questions before seeing how people similar to them were diagnosed. Users usually described between 3 and 4 symptoms (mean 3.2) in the health dialogue. The top first symptoms reported were headache (16%), back pain (13%), abdominal pain (8%), chest pain (8%) and rash (7%). The most common symptoms were insomnia, nausea, fatigue, and irritability. Women reported 10% more headache and 50% more abdominal pain than men. Men reported 17% more chest pain and 30% more back pain than women.

### 3.2 Diagnostic accuracy

In response to the follow up questions 82.4% of responders (895/1085) in MHS reported that the conditions presented at the end of K's health dialogues were in agreement with their doctor's final diagnosis. At Integrity Health Services, 85.4% of responses (111/130) were in agreement with the physicians’ final diagnosis.

## 4 Discussion

To date, more than one third of US adults use the internet to diagnose medical conditions.^[9]^ The variety of existing programs have tried to address the increasing needs of the public for up to date and accurate medical information. The common denominator of programs such as Ada and Babylon is that they compress, using different methods, information existing in medical textbooks into a rule engine and app.^[10]^ Typically, consensus statements by expert medical societies address single clinical situations and create updated guidelines in narrow fields of knowledge. The preliminary pilot conducted by us (see Appendix) clearly demonstrated that by and large the public does not receive the answers it asks for with the existing approaches. In contrast, the new K App did not create a system based on the rules of medical taxonomy, but rather a system that understands and follows the path a regular physician employs to approach her/his patient, on the basis of true experience and conversations with patients. With K's approach, the subject receives personalized view into how physicians have treated similar cases in real life, rather than general knowledge from textbooks, the algorithm investigates the specific case relative to similar cases. It is nonetheless important to stress that K does not provide medical recommendations, nor does it replace the physician. Rather, it gives the individuals information they can further discuss when visiting the physician. K's personalized and reliable information replaces the reliance on classical search engines.

By harnessing breakthrough artificial intelligence methods and Maccabi's vast data set, we were able to extract valuable data stored in both electronic medical records as well as unstructured physician notes during patients’ visits. For example, cardiovascular diseases, diabetes and hypertension remain common causes of death worldwide. Increased patient's knowledge through use of modern technology may aid in earlier detection of cardinal risk factors such as diabetes and hypertension.^[11–13]^

Potential limitations of this study need to be addressed. While K Health's application can address most adult primary care concerns, it has not yet been tested or validated for children. The machine learning is entirely based on patients who chose to seek medical attention, and whose experiences have been recorded in Maccabi's database. The machine was trained to mimic the experience of having a conversation with a doctor. The current App does not address PLM cases where the patient did not seek care, and this can theoretically create a bias that will need to be addressed in future studies. The question whether patient's sharing with his/her physician the result of the K App, may affect the doctor's final diagnosis should be considered. We do not believe that whether a patient mentioned the use of the K App to his/her physician constitutes a source of bias. It is not different from a patient sharing with the physician a media report or results of programs such as Ada and Babylon he/she has read about a symptom they have. These are all part of normal discourse between physicians and their patients. Trying to control for the sources of input the patients share with their physicians during visits- is outside the scope of the present study. Even if the source of information given by the patient (the K App or other) are affecting the physician's train of thought and final diagnosis- this does not constitute a source of bias.

While the program achieves very high approval rates by its users, its primary achievement is the 82% to 85% accuracy in identifying the condition that was later diagnosed by the personal physician in each individual case. Moreover, the machine learning algorithm continues to update itself with the feedback achieved in each case, improving K's performance with increasing use.

## Author contributions

**Conceptualization:** Gideon Koren, Ran Shaul, Varda Shalev.

**Data curation:** Jason Lockett, Ariel Leventhal.

**Formal analysis:** Ran Shaul, Allon Bloch, Daniel Souroujon.

**Funding acquisition:** Ran Shaul, Allon Bloch.

**Investigation:** Jason Lockett.

**Methodology:** Gideon Koren, Allon Bloch, Varda Shalev.

**Project administration:** Allon Bloch, Varda Shalev.

**Resources:** Allon Bloch.

**Software:** Ariel Leventhal, Daniel Souroujon.

**Validation:** Jason Lockett.

**Writing – original draft:** Gideon Koren.

**Writing – review & editing:** Gideon Koren, Ran Shaul, Allon Bloch, Daniel Souroujon, Varda Shalev.

Gideon Koren orcid: 0000-0002-9234-0875.

## Supplementary Material

Supplemental Digital Content
