# Supplementary material for: “A patient like me” – An algorithm-based program to inform patients on the likely conditions people with symptoms like theirs have
Source: Medicine (Baltimore). 2019 Oct 18;98(42):e17596. doi: 10.1097/MD.0000000000017596 (PMC6824689; doi:10.1097/MD.0000000000017596)
Supplement: Supplemental Digital Content [file medi-98-e17596-s001.docx]

**Appendix: preliminary survey**

The survey, conducted in December 2017, was based on a random sample of 500 adults , and the main findings indicated that the public, searching for a great deal of medical information online, believes that it is unreliable, and nonetheless often relies on it.

A total of 28% of respondents searched more than five times for online medical information, 75% searched twice or more in the past year, and only 5% of responders never searched for online medical information. Thirty percent conducted an online search following a media report or TV program, and 66% ran an online medical information search when they had experienced pain.

The main disadvantages identified by respondents were as follows:

1. The online search was not personally suited to their case (54%).

1. It was insufficiently professional (50%).
2. The information caused stress / anxiety (39%).
3. It generally presented severe or extreme options (38%).

In terms of precision, 54% of the survey responders found that the online data were erroneous, 66% concluded that the online diagnosis was far more severe than the problem was in reality, and 50% had such experiences more than once in the past year.

In numerous cases, such searches drove to action: One in 10 survey responders took medications or began medical treatment on their own initiative following an online search. 71% set an appointment with a doctor following an online search, 75% sought additional online information on their medical issue following a visit to the clinic or a physician’s diagnosis, and one in three testified that the online search undermined their confidence in the physician’s diagnosis.
